# Safer Prescribing and Care for the Elderly (SPACE): a cluster randomised controlled trial in general practice

**DOI:** 10.3399/BJGPO.2021.0129

**Published:** 2021-12-15

**Authors:** Katharine A Wallis, Carolyn Raina Elley, Simon A Moyes, Arier Lee, Joanna F Hikaka, Ngaire M Kerse

**Affiliations:** 1 Department of General Practice and Primary Health Care, School of Population Health, The University of Auckland, Auckland, New Zealand; 2 Primary Care Clinical Unit, Faculty of Medicine, The University of Queensland, Brisbane, Australia

**Keywords:** aged, anti-inflammatory agents, non-steroidal, drug-related side effects and adverse reactions, family medicine, multimorbidity, patient safety, polypharmacy, prescriptions, primary healthcare, general practice

## Abstract

**Background:**

Safer prescribing in general practice may help to decrease preventable adverse drug events (ADE) and related hospitalisations.

**Aim:**

To test the effect of the Safer Prescribing and Care for the Elderly (SPACE) intervention on high-risk prescribing of non-steroidal anti-inflammatory drugs (NSAIDs) and/or antiplatelet medicines and related hospitalisations.

**Design & setting:**

A pragmatic cluster randomised controlled trial in general practice. Participants were patients at increased risk of ADEs from NSAIDs and/or antiplatelet medicines at baseline. SPACE comprises automated search to generate for each GP a list of patients with high-risk prescribing; pharmacist outreach to provide education and one-on-one review of list with GP; and automated letter inviting patients to seek medication review with their GP.

**Method:**

The primary outcome was the difference in high-risk prescribing of NSAIDs and/or antiplatelet medicines at 6 months. Secondary outcomes were high-risk prescribing for gastrointestinal, renal, or cardiac ADEs separately, 12-month outcomes, and related ADE hospitalisations.

**Results:**

Thirty-nine practices were recruited with 205 GPs and 191 593 patients, of which 21 877 (11.4%) were participants. Of the participants, 1479 (6.8%) had high-risk prescribing. High-risk prescribing improved in both groups at 6 and 12 months compared with baseline. At 6 months, there was no significant difference between groups (odds ratio [OR] 0.99; 95% confidence intervals [CI] = 0.87 to 1.13) although SPACE improved more for gastrointestinal ADEs (OR 0.81; 95% CI = 0.68 to 0.96). At 12 months, the control group improved more (OR 1.29; 95% CI = 1.11 to 1.49). There was no significant difference for related hospitalisations.

**Conclusion:**

Further work is needed to identify scalable interventions that support safer prescribing in general practice. The use of automated search and feedback plus letter to patient warrants further exploration.

## How this fits in

It is not known how best to support safer prescribing in general practice. This trial tested a brief intervention comprising automated search, pharmacist delivered education and feedback, and automated letter to prompt patients to seek medicines review. The intervention had a partial, short-term effect. Brief interventions likely need repeating at regular intervals to achieve sustained improvement in general practice.

## Background

'Medication without harm' is a Global Patient Safety Challenge theme and top priority for the World Health Organization, which aims to reduce severe avoidable medication-related harm by 50% globally between 2017 and 2022.^1^ Most prescribing of ongoing medication occurs in general practice, making safer prescribing in this context important for protecting patient safety. ADEs in primary care are common, and they cause distress and burden health systems.^2–7^ About one-fifth of ADEs in primary care may be preventable through safer prescribing.^2^

Cardiovascular medications (including anti-platelet medicines) and NSAIDs account for up to one-third of serious ADEs in older age groups. These include upper gastrointestinal bleeding, kidney injury, and exacerbation of heart failure, and so are an important target for improvement.^2,7,8^ High-risk prescribing places patients at increased risk of ADEs, although it may be justified by the individual circumstances of the patient. People taking multiple medications are particularly at risk. Ethnic disparities for prescribing and ADEs exist. In New Zealand, Māori, and Pasifika people are more likely than other ethnic groups to be prescribed NSAIDs, and suffer associated ADEs at higher rates and younger ages.^9,10^

Trials of interventions to decrease high-risk prescribing in general practice have demonstrated improvements. The quality of evidence, however, is mixed, with many studies having short-term follow-up or interventions that are not readily scalable.^11–16^ One cluster randomised stepped wedge trial of a pharmacist-led intervention plus financial incentives found decreased high-risk prescribing of NSAIDs and related ADE hospital admissions; however, this trial did not have a true control group.^17^ Two recent systematic reviews found no high-quality evidence that interventions in general practice decrease ADEs or related hospitalisations, despite short-term improvements in prescribing.^15,18^

An intervention was developed to support safer prescribing in general practice, which can be applied to any prescribing topic (SPACE). A pilot study of SPACE showed promising results and good GP and patient acceptability.^13,19^ Then, the effectiveness of SPACE on high-risk prescribing in general practice for NSAIDs and/or anti-platelet medicines and related ADE hospitalisations was tested.^20^

## Method

### Study design and participants

**Figure 1.**
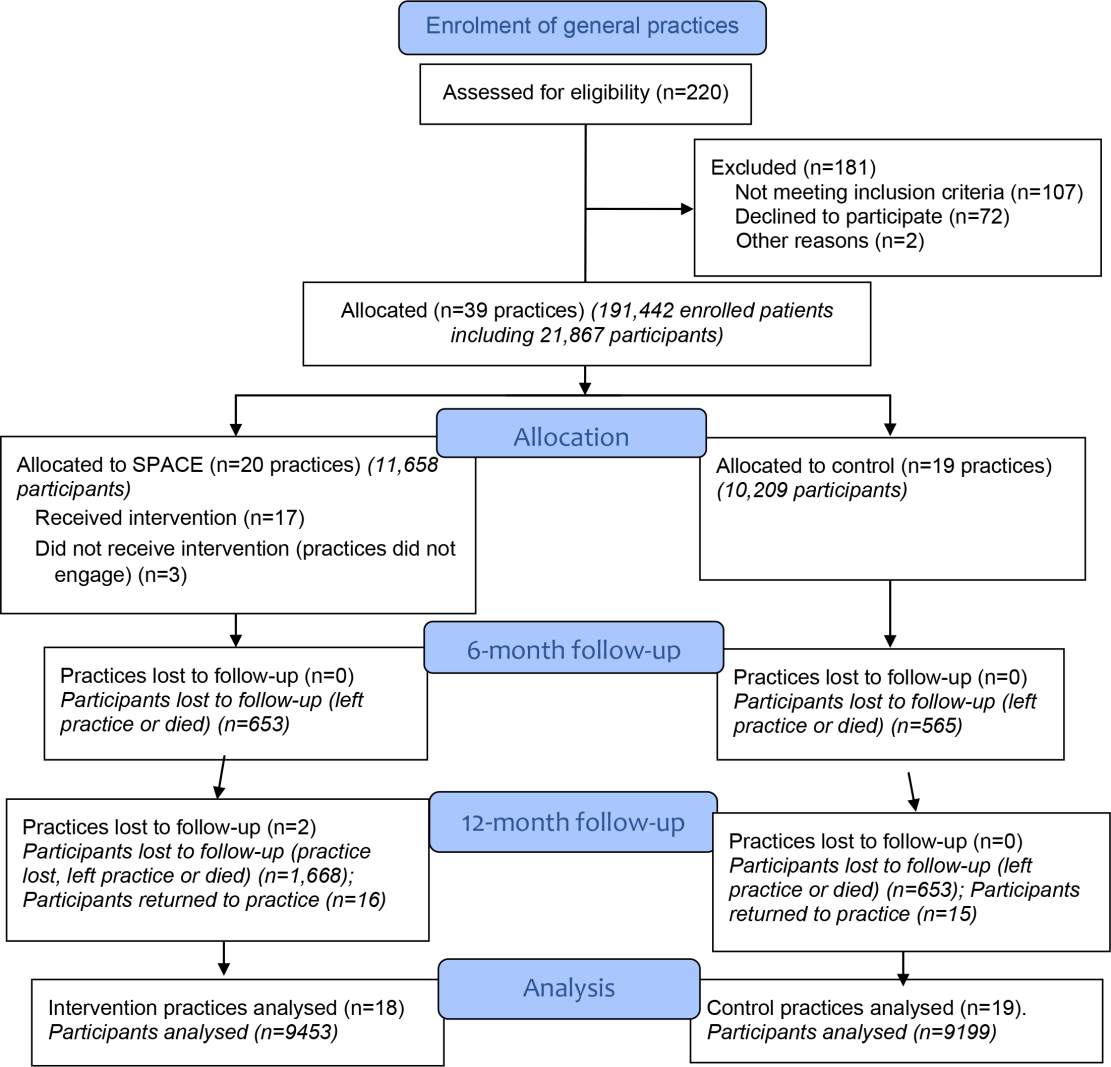
CONSORT diagram of SPACE cluster randomised controlled trial. CONSORT = Consolidated Standards of Reporting Trials. SPACE = Safer Prescribing and Care for the Elderly.

A pragmatic cluster randomised controlled trial design was used with the practice as the unit of randomisation (Figure 1).^20^ All GP practices in two regions of New Zealand were identified and eligible practices were invited via email and telephone to participate. Practices were not informed of the trial's high-risk prescribing topic. Eligibility was restricted to practices that used compatible electronic practice management systems and where all GPs consented to participate. Practices were excluded if they had participated in the SPACE pilot study or were participating in a non-trial initiative focusing on NSAID prescribing. Patients were included as participants if, at baseline, they were identified as at increased risk of gastrointestinal, renal, or cardiac ADEs from NSAIDs or anti-platelet medications (Table 1).^20^ Written consent for participation was obtained from all GPs, or from the practice manager on their behalf. As all data were anonymised and linked by encrypted national health identifier (NHI) before extraction, written consent was not required from patients.

**Table 1.** Participant inclusion criteria: patients at increased risk of ADEs with NSAIDs or antiplatelet medication at baseline

**Type of ADE**	**Any of the following clinical criteria**
Gastrointestinal bleed	Prior peptic ulcer diagnosis^a^
	Aged ≥75 years
	Aged ≥65 years and prescribed aspirin
	Prescribed oral anticoagulant
Renal impairment	Prescribed both renin-angiotensin system blocker and diuretic
	Chronic kidney disease (eGFR<60 at the most recent test)
Cardiac failure	Heart failure ever^a^

Prescribed medication is defined as any prescription for this category of medication in the previous 14 weeks, as identified from the electronic prescribing database of the participating practices. This time period is used because in New Zealand most medication is prescribed for a maximum of 12 weeks.

^a^Obtained general practice electronic disease coding from the participating practices.

ADE = adverse drug event. eGFR = estimated glomerular filtration rate. NSAID = non-steroidal anti-inflammatory drug.

### Randomisation and masking

Enrolled practices were stratified by region and practice size (small = 0–2999 enrolled patients; medium = 3000–7999; and large = 8000–14 999) and randomly assigned within strata to receive intervention or control in randomly occurring blocks of two and four. The random sequence was generated by a statistician not involved in recruitment or baseline data extraction. All outcomes and anonymised data extraction procedures were pre-specified and automated. Data at each time point were electronically extracted, de-identified, and sent by secure file to analysts (SM, CRE, and AL), all of whom were masked to allocation until after trial completion.

### SPACE intervention

The intervention comprised:

- automated search of practice records using pre-defined algorithms to identify and generate for each GP a list of patients with high-risk prescribing for NSAIDs and/or anti-platelet medications;
- outreach visit from the trial clinical advisory pharmacist to provide a 1-hour group educational session with GPs on the prescribing topic;
- to meet one-on-one with each GP, and support them in reviewing their list of patients and selecting for each patient an intended action from a tick-box in the computer software (‘Letter’; ‘No letter but review’; ‘No action’); and
- an automated letter from GPs to selected patients prompting patients to discuss their medicines at their next scheduled appointment.^13,19,20^

GPs were recompensed for their time with a NZ$100 gift voucher. All prescribing decisions were made as usual by GP and patient together. Control practices provided usual care.

### Outcome measures

Practice prescribing data were extracted using automated pre-defined algorithms at baseline, and 6 months and 12 months after allocation. The primary outcome was the difference between SPACE and control practices in the proportion of participants with high-risk prescribing of NSAID and/or antiplatelet medicines at 6 months, after adjusting for baseline and clustering by practice (Table 2). Secondary outcomes included proportions of participants at 6 months with high-risk prescribing of NSAID and/or antiplatelet medicines for gastrointestinal, renal, and cardiac ADEs separately; all outcomes at 12 months; and related hospitalisations for the 12 months following baseline compared with the previous 12 months (see Supplementary Table S7). Diagnostic codes and lab results were obtained from general practice electronic patient management systems. Prescribed medication was defined as any prescription for this category of medication in the practice electronic prescribing database in the previous 14 weeks. This time period was used because in New Zealand patients must obtain repeat prescriptions for ongoing medications every 12 weeks. Anonymised patient-level demographic and clinical data were extracted and linked over time by encrypted NHI. National level hospitalisation and mortality data were also linked by encrypted NHI to identify hospitalisation outcomes.

**Table 2.** High-risk prescribing outcome measures

**ADE type**	**High-risk prescribing**
Gastrointestinal	NSAID or aspirin without gastroprotection in patient with prior peptic ulcer^a^ ***or***
	NSAID without gastroprotection in patient aged ≥75 years ***or***
	NSAID without gastroprotection in patient aged ≥65 years taking aspirin ***or***
	Clopidogrel without gastroprotection in patient aged ≥65 years taking aspirin ***or***
	NSAID without gastroprotection in patient taking an oral anticoagulant ***or***
	Aspirin or clopidogrel without gastroprotection in patient taking an oral anticoagulant
Renal	NSAID in patient taking both renin-angiotensin system blocker and diuretic ***or***
	NSAID in patient with chronic kidney disease (eGFR <60 at the most recent test)
Cardiac	NSAID in patient with history of heart failure^a^
Combined	Any of the above criteria

Prescribed medication is defined as any prescription for this category of medication in the previous 14 weeks, as identified from the electronic prescribing database of the participating practices. This time period is used because in New Zealand most medication is prescribed for a maximum of 12 weeks.

^a^Obtained general practice electronic disease coding from the participating practices.

ADE = adverse drug event. eGFR = estimated glomerular filtration rate. NSAID = non-steroidal anti-inflammatory drug.

### Sample size calculations and statistical analysis

The sample size calculation was based on previous trials demonstrating a clinically relevant 25–45% relative risk reduction in the proportion of high-risk NSAID or antiplatelet prescribing.^12,17^ An average of 200 participants per practice and an intracluster correlation coefficient (ICC) of 4.68 × 10^–7^ for the primary outcome was estimated.^12^ Based on local feasibility study data, an 8% high-risk prescribing rate was assumed. Assuming approximately 12% of patients would be lost to follow-up over the 12-month study period, data from 8000 patients from 40 practices (20 practices in each group) with an average of 200 participants per practice were required to detect a statistically significant difference of 6% in the intervention group and 8% in the control group of high-risk prescribing at follow-up (*P* = 0.9, alpha = 0.05).

Statistical analyses were performed according to the intention-to-treat (ITT) principle, with the use of mixed-effect models to account for clustering in the data. Primary analysis was by practice allocation. The proportions of participants with high-risk prescribing were analysed using random-effects logistic regression, with the individual as the unit of analysis and the practice included as the random effect to control for the effects of clustering. GLIMMIX with group-by-time interaction was used to assess the overall difference between intervention and control at 6 and 12 months. The model adjusted for the stratification factors including practice location (region A or B) and practice size (small, medium, or large).

An inadvertent transcription error inverted the random group assignment of six practices before allocation (I, C, I, C, I, C instead of C, I, C, I, C, I where ‘I’ is intervention and ‘C’ control) which was not apparent until the end of the trial. Analysis by allocation is presented in the Results section below. Per-protocol analysis and analysis by original random group assignment are provided in the online Supplementary Appendix.

## Results

### Practices and participants

Of the 220 general practices identified, 110 fulfilled inclusion criteria, of which 39 (35.5%) agreed to participate and were enrolled between April 2018 and July 2019 (Figure 1). There were 14 small, 18 medium, and seven large practices. Most practices not fulfilling inclusion criteria were either participating in the non-trial NSAID prescribing initiative, did not use compatible practice management systems, or had participated in the SPACE pilot study. The main reason eligible practices gave for declining was busyness.

There were 191 442 patients registered in the 39 trial practices. Of these, 21 867 (11.4%) were identified as participants (at increased risk of ADEs from NSAIDs or antiplatelet medications), of whom 1479 (6.8%) had high-risk prescribing over the previous 14 weeks (Figure 1). Demographic and clinical characteristics of participants were similar (Table 3), although high-risk prescribing rates at baseline were higher in SPACE (7.1%) than in control practices (6.4%) (Table 4). By 6 months, *n* = 1218/21 867 (5.6%) participants were lost to follow-up (Figure 1). By 12 months, two large intervention practices had changed data management systems, resulting in a loss to follow-up of *n* = 3215/21 867 (14.7%) participants.

**Table 3.** Baseline demographic and clinical characteristics of study participants

	**SPACE (*n* = 11 658**)	**Control (*n* = 10 209**)	**Total (*n* = 21 867**)
Mean age, years (SD)	73.6 (11.9)	73.1 (12.0)	73.4 (12.0)
Female, *n* (%)	5997 (51%)	5376 (53%)	11 373 (52%)
Ethnicity, *n* (%)			
NZ European	7334 (63%)	7008 (69%)	14 342 (66%)
Other European	1674 (14%)	902 (9%)	2576 (12%)
NZ Maori	732 (6%)	902 (9%)	1634 (7%)
Pasifika	459 (4%)	451 (4%)	910 (4%)
East Asian	756 (6%)	374 (4%)	1130 (5%)
Indian	341 (3%)	308 (3%)	649 (3%)
Other	362 (3%)	264 (3%)	626 (3%)
Number of long-term medications, mean (SD)	5.1 (3.7)	5.6 (3.8)	5.3 (3.8)

NZ = New Zealand. SD = standard deviation. SPACE = Safer Prescribing and Care for the Elderly.

**Table 4.** Changes in rate of high-risk prescribing to participants in SPACE versus control practices at 6 and 12 months

	**Baseline**		**6-month *n*/*N* (%)**		**12-month *n*/*N* (%)**		**OR (6 months)** ^a^(**95% CI**)	***P* value**	**OR (12 months)^a^** (**95% CI**)	***P* value**
	**SPACE**	**Control**	**SPACE**	**Control**	**SPACE**	**Control**				
**Primary outcome**										
Combined risk factor^b^	828/11 658 (7.1%)	651/10 209 (6.4%)	638/11 005 (5.8%)	537/9644 (5.6%)	538/9453 (5.7%)	419/9199 (4.6%)	0.99 (0.87–1.13)	0.9	1.29(1.11–1.49)	0.001
**Secondary outcomes**										
Gastrointestinal	427/8711 (4.9%)	334/7465 (4.5%)	313/7894 (4.0%)	301/6782 (4.4%)	232/5046 (4.6%)	208/4489 (4.6%)	0.81 (0.68–0.96)	0.02	0.91(0.74–1.11)	0.4
Renal	503/6268 (8.0%)	372/5588 (6.7%)	365/5124 (7.1%)	262/4477 (5.9%)	314/4318 (7.3%)	208/3988 (5.2%)	1.16 (0.96–1.39)	0.1	1.35(1.10–1.65)	0.004
Heart failure	21/613 (3.4%)	29/627 (4.6%)	19/557 (3.4%)	14/558 (2.5%)	17/441 (3.9%)	20/508 (3.9%)	1.84 (0.87–3.89)	0.1	0.89(0.44–1.78)	0.7

^a^Adjusted for clustering by practice. ^b^Gastrointestinal, renal, or cardiac ‘high-risk’ prescribing of NSAIDs and/or anticoagulant medication. OR = odds ratio. SPACE = Safer Prescribing and Care for the Elderly.

### Intervention delivery

There were often delays between baseline measures, group education, and one-on-one list review — ranging from 1 week to 6 months — mostly because GPs were 'too busy'. Often, the automated search was re-run to generate an up-to-date list of patients with high-risk prescribing for one-on-one review. In three intervention practices (two large and one medium), there was minimal or no engagement with the intervention (Supplementary Table S1); one practice did not receive the group education, and only one of 15 GPs agreed to meet with the pharmacist for the one-on-one list review. These three intervention practices included *n* = 254/828 (31%) participants with high-risk prescribing at baseline. Poor engagement resulted in the trial pharmacist having one-on-one sessions with only 70% of GPs and reviewing only 416 of the 683 participants (61%) identified at intervention delivery as having high-risk prescribing (Supplementary Table S1).

### GP tick-box selection (intended action)

The GP tick-box selections for the 416 participants reviewed were ‘Letter’ (*n* = 97, 23%); ‘No letter but review’ (*n* = 151, 36%); and ‘No action’ (*n* = 168, 40%). The reasons for ‘No letter but review’ included GP concern about upsetting the patient; patient not understanding written English; patient due to be seen soon; or GP preference to leave themselves a note. The reasons for ‘No action’ were mostly that the high-risk prescribing had already ceased (short-course or topical NSAIDs, proton pump inhibitors had been initiated, renal function had returned to normal); the patient had moved practice; the GP considered the individual circumstances of the patient justified the high-risk prescribing; or the GP did not agree that the prescribing was high-risk (Supplementary Table S1).

### Prescribing outcomes

High-risk prescribing decreased in both SPACE and control practices at 6 and 12 months compared to baseline as shown in Table 4. At 6 months, there was no significant difference in high-risk prescribing between groups (OR = 0.99; 95% CI = 0.87 to 1.13), although high-risk prescribing for gastrointestinal ADEs had improved significantly more in SPACE practices (OR = 0.81; 95% CI = 0.68 to 0.96) (Table 4). At 12 months, high-risk prescribing had improved more in the control than the SPACE group overall (OR = 1.29; 95% CI = 1.11 to 1.49) and for renal ADEs (OR = 1.35; 95% CI = 1.10 to 1.65). There was no significant difference in the outcomes between ethnic groups.

The per-protocol analysis showed that at 6 months there was no significant difference in high-risk prescribing overall (OR = 0.94; 95% CI = 0.82 to 1.08), although high-risk prescribing for gastrointestinal ADEs had improved significantly more in the SPACE group (OR = 0.76; 95% CI = 0.64 to 0.92), which was sustained at 12 months (OR = 0.75; 95% CI = 0.64 to 0.92) (Supplementary Table S2).

Analysis by original random group assignment showed the SPACE group was significantly more likely to have high-risk prescribing than the control group at 6 months (OR = 1.21; 95% CI = 1.06 to 1.38) (Supplementary Table S3).

### Hospitalisations

There was no significant difference between the groups for total hospitalisations or for related gastrointestinal, renal, or cardiac ADE hospitalisations in the 12 months following baseline adjusting for the 12 months before baseline (Table 5 and Supplementary Tables S4–S6).

**Table 5.** Number of participants hospitalised with related diagnoses in SPACE compared with control groups in the 12 months following baseline, adjusting for hospitalisations in the 12 months before baseline, patient age at baseline, and number of prescribed medications at baseline

	12 months before baseline, *n*/*N* (%)		12 months after baseline, *n*/*N* (%)		OR (**95% CI)**	*P* value
	SPACE	Control	SPACE	Control		
Combined risk factor	350/11 658 (3.0%)	359/10 209 (3.5%)	371/11 658 (3.2%)	364/10 209 (3.6%)	0.96 (0.82–1.11)	0.5^a^
Gastrointestinal	116/8711 (1.3%)	116/7465 (1.6%)	112/8711 (1.2%)	99/7465 (1.3%)	1.03 (0.78–1.35)	0.9
Renal	76/6268 (1.2%)	75/5588 (1.3%)	77/6268 (1.2%)	86/5588 (1.5%)	0.84 (0.62–1.15)	0.3
Heart failure	90/613 (14.7%)	76/627 (12.1%)	56/613 (9.1%)	66/627 (10.5%)	0.85 (0.57–1.25)	0.4

^a^Owing to the small number of hospitalisations in each practice, clustering could not be used in any model except this one. When clustering, sex and ethnicity were included in the model the P value was 0.3.

OR = odds ratio. SPACE = Safer Prescribing and Care for the Elderly.

## Discussion

### Summary

In this pragmatic trial in general practice, high-risk prescribing of NSAIDs and/or antiplatelet medicines improved in both groups at 6 and 12 months compared with baseline. This may have been owing to a general increase in awareness of the prescribing topic in the GP community. There was no significant difference between groups at 6 months in high-risk prescribing overall, although the SPACE group improved more for gastrointestinal ADEs. This improvement was not sustained at 12 months, when high-risk prescribing had improved more in the control group. There was no significant difference between groups in related ADE hospitalisations. The CONSORT-Equity extension of 2017 encourages the investigation of intervention effects in people experiencing social disadvantage.^21^ No ethnic differences in outcomes were found, although the trial was not powered to detect this.

A possible explanation for the partial, short-term effect of SPACE may be that improving prescribing for gastrointestinal ADEs involves starting medication (proton pump inhibitor) while improving prescribing for renal and cardiac ADEs requires stopping medication, which GPs can find more challenging.^22^ Given the time pressures and multiple competing demands in general practice it is not easy for interventions to produce lasting improvement. Brief interventions likely need repeating at regular intervals to achieve sustained improvement, balancing affordability and scalability.

### Strengths and limitations

The strengths of this study include pragmatic trial design, and a scalable intervention for use in general practice that may be applied to any prescribing topic, which includes novel elements of pharmacist supporting GPs to review a list of patients and select some for an automated letter prompting them to seek medicines review. Important limitations include lower than expected engagement and uptake of the intervention. For one-third of SPACE participants, practices did not engage or there was minimal engagement, making it difficult to show an effect. For practices that did engage, it was often difficult for the trial pharmacist to secure time with GPs, delaying intervention delivery. Unlike in the SPACE pilot study, the trial pharmacist was not known to practices and GPs which may explain the difficulties.^13^ A further limitation was that some practices enrolled in the contemporaneous prescribing initiative during follow-up, which confounded the results but reflects the real-world conditions in which the study was conducted. A further limitation was the transcription error, where one block of six practices were allocated to the inverse of the random group assignment generated. ITT analysis is presented by original random group assignment in the appendices, although this analysis risks a type two error (introducing bias towards the null hypothesis).

### Comparison with existing literature

This study's findings are consistent with previous research investigating ways to improve prescribing in general practice, which had limited success.^15,18,23–29^ Complex interventions combining educational outreach visits, audit, and feedback can work,^30,31^ as can interventions involving pharmacists in general practices,^12,28,32,33^ especially when combined with financial incentives.^17^ Interventions that include empowering patients have also shown promise.^34^ An intervention that included seven prescribing audits, seven face-to-face or group sessions, and then sessions every 4 months showed a lasting effect on high-risk prescribing.^11^

### Implications for research

Further work is needed to identify scalable interventions that support safer prescribing in general practice in the longer term. The role of practice-based clinical advisory pharmacists and the use of automated searches to generate lists of at-risk patients for each GP and letters to patients to prompt medication review warrant further exploration.

## Trial registration

ACTRN12618000034235, January 2018.
